# Leishmanicidal Activity of a Hydrazone Derivative Loaded into Nanocarrier Systems

**DOI:** 10.1021/acsomega.5c13239

**Published:** 2026-05-27

**Authors:** Juliana B. Nunes, Thalles H. F. de Souza, Amanda S. Lima, Clara O. C. Lopes, Isabelly F. Ferraz de Souza, Raíne P. Amaral, Gislaine R. Pereira, Fábio A. Colombo, Eduardo C. Figueiredo, Luciana Azevedo, Luiz F. Leomil Coelho, Luis F. Cunha dos Reis, Lídia M. Lima, Marcos J. Marques

**Affiliations:** † Biomedical Sciences Institute, Pathology and Parasitology Department, 74347 Alfenas Federal University (UNIFAL-MG), 37.130-001 Alfenas, Brazil; ‡ Nutrition School, Alfenas Federal University (UNIFAL-MG), 37.130-001 Alfenas, Brazil; § School of Pharmaceutical Sciences Alfenas Federal University (UNIFAL-MG), 37.130-001 Alfenas, Brazil; ∥ Biomedical Sciences Institute, Microbiology and Immunology Department, Alfenas Federal University (UNIFAL-MG), 37.130-001 Alfenas, Brazil; ⊥ Biomedical Sciences Institute, Structural Biology Department, Alfenas Federal University (UNIFAL-MG), 37.130-001 Alfenas, Brazil; # Faculty of Pharmacy, 28125 Federal University of Rio de Janeiro (UFRJ), Av. Carlos Chagas Filho, 373, Cidade Universitária, Rio de Janeiro, Rio de Janeiro 21941-902, Brazil

## Abstract

Visceral leishmaniasis (VL) is a neglected parasitic disease whose treatment is limited by parasite resistance, drug toxicity, and high costs. Seeking safer and more effective therapies, we evaluated the hydrazone derivative LASSBio-1736, a cysteine protease inhibitor hydrazone derivative, which demonstrated plasma stability and low initial hepatic and renal toxicity, focusing on its oral delivery in bovine serum albumin nanoparticles (LASSBio-1736n). An HPLC–UV method was validated for quantitative analysis in biological samples, showing high sensitivity, precision, and linearity. Standardized in vitro digestion (INFOGEST) and Caco-2 assays demonstrated enhanced bioaccessibility and intestinal permeability of the nanoformulation. In the INFOGEST model, LASSBio-1736n maintained structural integrity and drug content, with a 239.7% increase between gastric and intestinal phases compared to the free compound (LASSBio-1736f). Caco-2 assays further confirmed higher apparent permeability, supporting improved oral absorption. In vivo efficacy was evaluated in golden hamsters (*Mesocricetus auratus*) infected with *Leishmania* (L.) *infantum chagasi*. Animals treated orally with LASSBio-1736n (12.5 mg/kg/day for 10 days) showed a significant reduction (85.48%) in splenic parasite burden compared to Glucantime. This effect was associated with cytokine modulation (increased IL-10 and reduced IFN-γ) and histopathological improvement, including reduced granuloma area, balanced parenchyma/interstitium ratio, and normalized hepatocyte glycogen distribution. LASSBio-1736f showed milder effects, whereas Glucantime induced a stronger pro-inflammatory response. Overall, these findings demonstrate that albumin-based nanocarriers enhance the in vivo efficacy of LASSBio-1736 and support its development as a promising orally deliverable therapy for VL.

## Graphical abstract

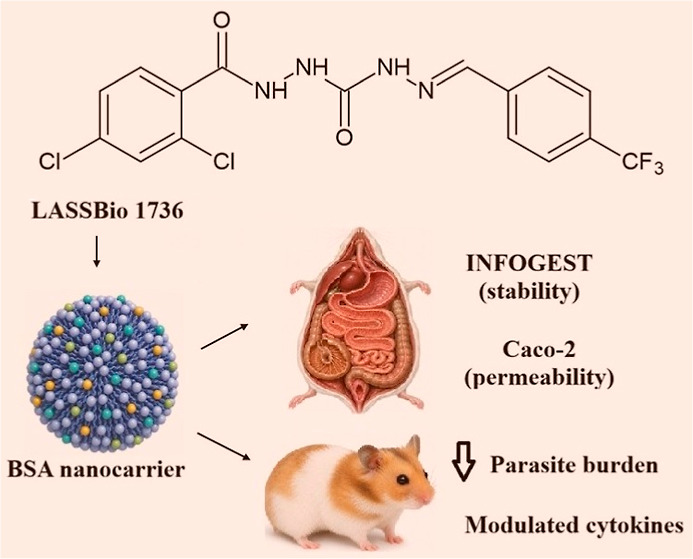

## Introduction

Leishmaniasis is one of the most important neglected tropical diseases, encompassing cutaneous and visceral clinical forms. Cutaneous manifestations cause disfiguring skin lesions and social stigma, whereas visceral leishmaniasis (VL) primarily affects internal organs and can be fatal if left untreated.^1^ Consequently, effective therapy is critical to reduce morbidity, prevent complications, and improve patients’ quality of life while supporting disease control at the population level. Despite this need, global investments in innovative diagnostics, vaccines, and therapeutic strategies remain limited.^2^

The limited pharmacotherapeutic options currently available for leishmaniasis show limited efficacy and the emergence of parasite resistance.^3^ Moreover, many of these drugs present significant toxicity and are often poorly tolerated.^4^ These limitations highlight the urgent need for safer, more effective therapies, including those based on public–private partnerships.^5^

Within this context, the hydrazone derivative (*E*)-1-(4-trifluoromethylbenzylidene)-5-(2,4-dichlorobenzoyl)­carbonohydrazide (LASSBio-1736; logP 4.29) has demonstrated potent in vitro leishmanicidal activity against *Leishmania* (L.) *braziliensis* and *Leishmania* *amazonensis*, as well as in vivo efficacy in experimental cutaneous leishmaniasis.^6^ Considering these findings, a less lipophilic isostere, LASSBio-1491, exhibited improved pharmacokinetic properties compatible with once-daily dosing. This compound also demonstrated superior in vivo activity compared with Glucantime in *Leishmania major* infection models, without detectable cytotoxicity in mammalian cells.^7^

Nanotechnology has emerged as a promising strategy to overcome key limitations in drug delivery, as nanoscale carriers could improve drug solubility and bioavailability, reduce toxicity, and enhance therapeutic outcomes.^8^ Nanoparticle-based delivery systems may further improve antileishmanial therapy by enabling controlled drug release and targeted tissue distribution.^9^ Bovine serum albumin (BSA), a natural, biodegradable polymer, has been widely investigated as a drug carrier,^10^ and BSA nanoparticles have been successfully applied to experimental leishmaniasis, including as carriers for curcumin.^11^ Given the low aqueous solubility and limited oral bioavailability (∼12%) of LASSBio-1736,^6^ nano- and micro-structured formulations may provide significant pharmacological advantages to this compound.

Therefore, in this study, we developed and characterized BSA nanoparticles containing the (*E*)-1-(4-trifluoromethylbenzylidene)-5-(2,4-dichlorobenzoyl)­carbonohydrazide (LASSBio-1736n). Physicochemical characterization included particle size, ζ-potential, and polydispersity index (PDI). In addition, our group assessed in vitro gastrointestinal stability using a standardized INFOGEST digestion model and evaluated intestinal permeability in Caco-2 cell monolayers. Finally, the therapeutic potential of LASSBio-1736n or LASSBio-1736f (without BSA) was investigated in a hamster (*Mesocricetus auratus*) model of VL by quantifying parasite burden, cytokine expression, and histopathological alterations in the spleen and liver.

## Results and Discussion

BSA nanoparticles have been extensively investigated as drug delivery systems for a wide range of therapeutic applications, including the treatment of muscular trichinellosis,^12,13^ anticancer and antibacterial therapies,^14^ and even vaccine development strategies.^15,16^ In the context of leishmaniasis, BSA-based carriers have shown an improvement in leishmanicidal activity while reducing compound-related toxicity in in vitro and in vivo experimental models.^11,17^

In a recent study, curcumin-loaded BSA nanoparticles combined with photodynamic therapy were efficiently internalized by infected macrophages and significantly reduced infection by three *Leishmania* species (*Leishmania braziliensis*, *L. major*, and *L. amazonensis*) in vitro.^11^ Similarly, Casa et al. (2018)^17^ reported that even though it is less potent than amphotericin deoxycholate, BSA-encapsulated amphotericin markedly lowered toxicity in both in vitro and in vivo settings.

Meanwhile, in the present research, BSA nanoparticles were successfully loaded with the hydrazone derivative (*E*)-1-(4-trifluoromethylbenzylidene)-5-(2,4-dichlorobenzoyl)­carbonohydrazide (LASSBio-1736n). The formulation resulted in an encapsulation efficiency (EE) of 96.34%, indicating that nearly all of the drug added was associated with the nanoparticles.

The drug loading capacity (LC), which represents the amount of encapsulated compound relative to the polymer mass, reached 4.34%. This relatively low LC reflects the small amount of LASSBio-1736 used during preparation (1 mg of compound per 20 mg of BSA), indicating that the formulation was not optimized for maximum drug loading. Increasing the initial drug-to-polymer ratio may represent a straightforward strategy for enhancing LC. In addition, further optimization approaches, such as adjusting cross-linking conditions, solvent composition, or nanoparticle preparation parameters, could be explored to improve loading efficiency without compromising nanoparticle stability. Figure S1 presents the calibration curve obtained by HPLC, which was used to quantify LASSBio-1736 for EE and LC determination.

The size and surface charge of BSA nanoparticles, with or without LASSBio-1736, are presented in Table 1 and Figure 1. The negative ζ-potential observed (Table 1) for LASSBio-1736n indicates high colloidal stability of the nanoparticle suspension, as electrostatic repulsion between particles helps prevent aggregation.

**Table 1:** Size, Zeta Potential, and PDI Analysis of Empty Nanoparticles (BSAn) and Those Containing the Compound LASSBio-1736 (LASSBio-1736n) Using the Scanning Electron Microscopy (SEM), ZetaSizer, and ZetaView Instruments

NP	size (nm) (SEM)	zeta potential (mV) (ZetaView)	PDI (ZetaSizer)
BSAn	155.9 ± 125	–27.4 ± 1.14	0.272
LASSBio-1736n	148.3 ± 97.6	–40.12 ± 0.93	0.252

**Figure 1:**
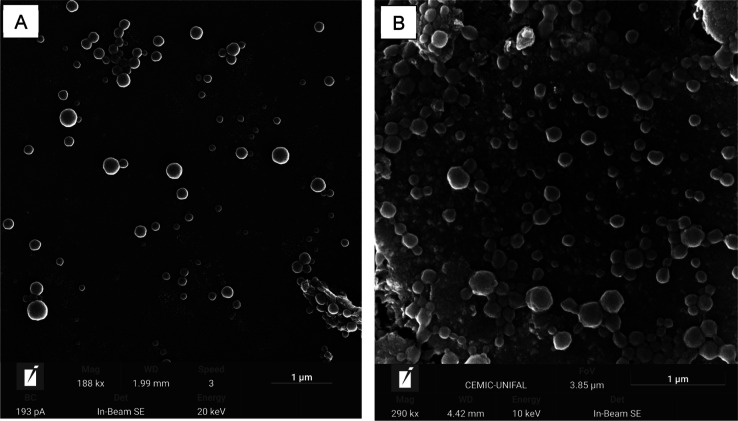
Scanning electron microscopy (SEM) images showing the size and morphology of LASSBio-1736n (A) and empty albumin nanoparticles (BSAn) (B).

To evaluate oral stability, LASSBio-1736n and LASSBio-1736f were subjected to the standardized INFOGEST in vitro digestion model (oral, gastric, and intestinal phases in sequence). The presence of LASSBio-1736 after each digestion step was monitored by UHPLC–MS through detection of the characteristic ion at *m*/*z* 419, corresponding to the protonated molecule of the compound. This approach confirmed its identity and enabled the evaluation of compound stability throughout the simulated digestion process (Figure 2). Calibration curves were established (Figure S2), enabling quantification of residual compound after each digestion phase (Table S1).

**Figure 2:**
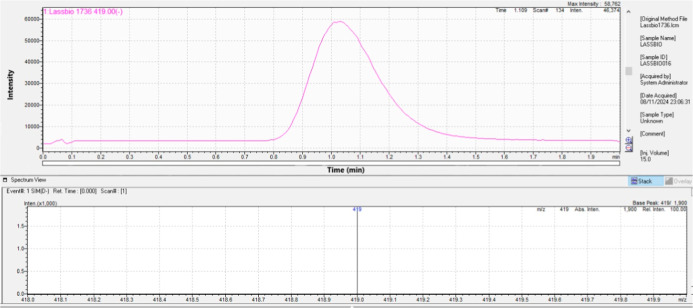
UHPLC–MS detection of LASSBio-1736 (*m*/*z* 419).

Within the INFOGEST-simulated digestion model, LASSBio-1736n maintained its structural integrity and preserved drug content, demonstrating superior stability and bioaccessibility throughout gastrointestinal transit. When compared with LASSBio-1736f, LASSBio-1736n showed a +239.7% relative variation between the gastric and intestinal phases, corresponding to an approximately 3.4-fold higher residual concentration of LASSBio-1736 in the intestinal phase. These findings showed that BSA encapsulation effectively preserved the stability of LASSBio-1736 during simulated gastrointestinal digestion (Figure 3 and Table S1). The marked increase suggests that the nanoparticle system enhanced compound retention by mitigating acidic degradation and enzymatic hydrolysis in the gastric phase, resulting in a higher recovery under the neutral conditions of the intestinal phase.

**Figure 3:**
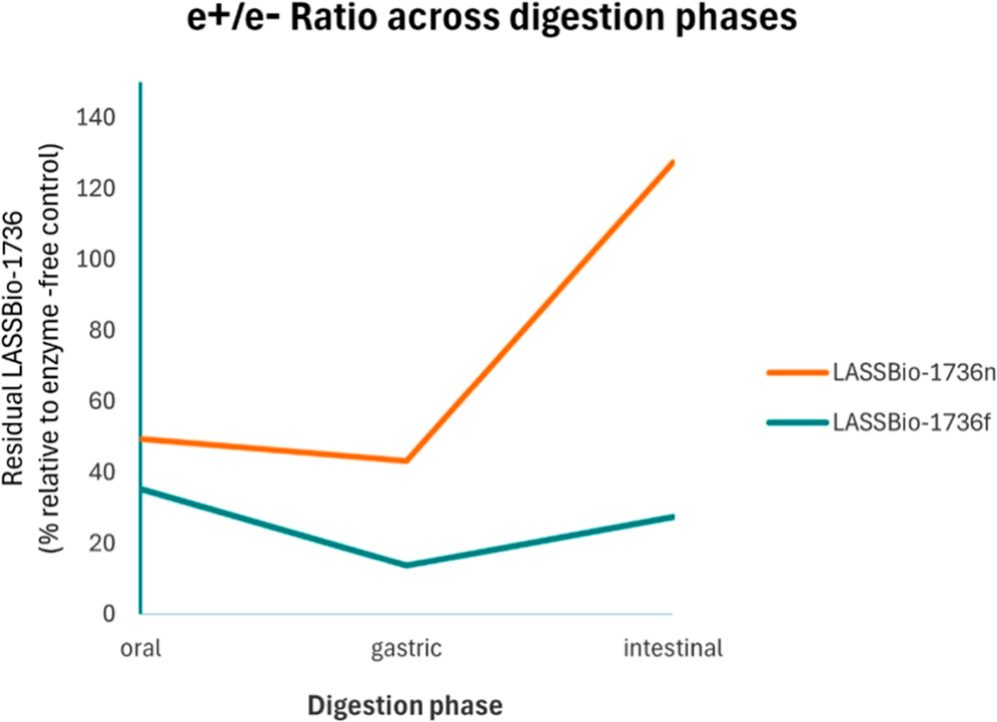
Residual LASSBio-1736 during simulated gastrointestinal digestion. Data are expressed as the percentage of compound detected by UHPLC–MS in the presence of digestive enzymes relative to enzyme-free controls (e+/e– ratio). Oral, gastric, and intestinal correspond to the sequential phases of the standardized INFOGEST digestion model. LASSBio-1736f represents the free compound, whereas LASSBio-1736n represents the nanoparticle formulation.

Wang et al. (2025)^18^ reported a similar protective effect, demonstrating that curcumin nanoparticles maintained high gastrointestinal stability, reaching ∼92% when incorporated into nanoemulsions, whereas free curcumin underwent significant aggregation and loss in the gastric phase. In both systems, nanoencapsulation played a key role in improving the bioaccessibility of hydrophobic compounds by promoting their incorporation into mixed micelles during intestinal digestion.

Therefore, the behavior observed for nanoparticulated LASSBio-1736 aligns with the concept that nanocarriers provide structural stability across different digestion phases. As a result, it facilitates compound preservation and controlled release in the small intestine. This enhancement in intestinal recovery highlights the potential of the formulation to improve the bioavailability of the poorly soluble compounds.

Permeation of free and nanoencapsulated LASSBio-1736 across differentiated Caco-2 monolayers was detected after 30 min (Table 2). Figure S3 presents the calibration curve obtained by HPLC, which was used to quantify LASSBio-1736 in the permeation experiment. After 30 min, LASSBio-1736n exhibited significantly higher apparent permeability in the apical-to-basolateral (A → B) direction (*p* < 0.01). In contrast, in the basolateral-to-apical (B → A) direction, LASSBio-1736f exhibited higher permeability than the nanoparticulate formulation (*p* < 0.05). These data indicate that the nanocarrier provides a pharmacotechnical advantage that may enhance oral bioavailability.

**Table 2:** LASSBio-1736n (Nanoparticulated) and LASSBio-1736f (Free) Permeated across Caco-2 Monolayers after 30 min (μg/L), and Their Respective Permeation Percentages

		direction	
compound	time (min)	A–B	B–A
LASSBio-1736f	0	4418.38 ± 47.31	1612.55 ± 107.83
	30	84.72 ± 133.95	216.78 ± 20.61
	%	1.9	13.4
LASSBio-1736n	0	138.72 ± 68.88	4746.47 ± 12.21
	30	128.50 ± 70.61	22.75 ± 129.08
	%	92.6	0.5
*p*-value^a^		<0.01	<0.05

a Data are presented as mean ± standard deviation (SD) (*n* = 3). Due to non-normal data distribution and high variability, statistical comparisons were performed using the Mann–Whitney test.

After 30 min in the Caco-2 permeability assay, LASSBio-1736f showed a marked reduction in apical-to-basolateral transport, retaining only 1.9% of its initial concentration. In contrast, the nanoparticulated formulation maintained 92.6% of the initial permeation in the same direction, indicating pronounced stability and sustained transport. Conversely, basolateral-to-apical efflux (B → A) was markedly lower for the nanoparticulated system (0.5%) than for the free compound (13.4%), suggesting that nanoencapsulation reduces efflux while promoting intracellular retention or controlled drug release. Overall, these results indicate that the nanoparticulate delivery system modulates the bidirectional permeability of LASSBio-1736, enhancing its apical-to-basolateral stability and potentially improving intestinal absorption and bioavailability.

It is important to note that the measured signal in this assay represents the total detectable compound after transport, which may include contributions from intact nanoparticles, released drugs, and membrane-associated fractions. Therefore, the high retention values observed for the nanoparticle formulation likely reflect a combination of enhanced cellular interaction, intracellular retention, metabolism by enzymatic systems present in Caco-2 cells, and controlled drug release rather than solely transcellular permeation of the free compound. This behavior is consistent with the known properties of nanoparticle-based delivery systems and highlights the limitations of the Caco-2 model in distinguishing between compound transport and cellular processing.^19^ These factors should be considered when interpreting the apparent permeability results.

In a study by Moraes et al. (2016),^20^ a robust HPLC-UV analytical method was developed and validated for the detection and quantification of LASSBio-1736 in biological matrices, thereby enabling subsequent preclinical pharmacokinetic investigations. Accordingly, plasma pharmacokinetics, protein binding, and tissue distribution of LASSBio-1736 were investigated in Wistar rats. Using different routes of administration, the compound exhibited a large volume of distribution, a prolonged half-life, low systemic clearance, and efficient penetration into key target tissues, including the liver, spleen, and skin.^21^ The administration routes evaluated may be applied in future preclinical trials involving infections with different *Leishmania* species.

The cytotoxicity of LASSBio-1736 toward mammalian cells has been previously evaluated in murine J774 macrophages using a lactate dehydrogenase (LDH)-based viability assay. No cytotoxic effects were observed at concentrations up to 100 μM, indicating a CC_50_ value above this threshold.^21^ In addition, studies on related cysteine protease inhibitor scaffolds have reported low cytotoxicity of LASSBio-1736 and structurally related derivatives in J774. A1 macrophages, reinforcing its favorable safety profile.^22^ Based on previously reported IC_50_ values against intracellular amastigote forms of *L. braziliensis* (5.3 μM) and *L. amazonensis* (84 μM), the estimated selectivity indices are therefore greater than 18.9 and 1.2, respectively.^22^ These results support the selective antileishmanial activity of LASSBio-1736 and reinforce its potential as a promising lead compound for further pharmacological development.

According to previous studies with LASSBio-1736 (log P 4.54), a less lipophilic isostere named LASSBio-1491 (log P 2.14) was developed.^7^ This derivative demonstrated leishmanicidal activity in both in vitro and in vivo models and exhibited high permeability, improved aqueous solubility, prolonged plasma and microsomal half-lives, and low in vitro systemic clearance. Overall, these properties indicate a favorable pharmacokinetic profile compatible with once-daily dosing and support the potential application of LASSBio-1491 for the treatment of cutaneous leishmaniasis caused by *L. major*.

Importantly, the in vitro pharmacokinetic data indicate a favorable profile for LASSBio-1736, including high plasma stability, low metabolic conversion, and prolonged half-life. These features are consistent with previous reports demonstrating low systemic clearance and the absence of preliminary hepatic or renal toxicity signals for this compound.^21^ Although detailed long-term toxicity and in vivo pharmacokinetic studies were not within the scope of the present work, and no acute toxicity evaluation was performed in the current in vivo model, the available data support the potential of LASSBio-1736 as a promising candidate for further preclinical development.

In the present study, the therapeutic potential of LASSBio-1736 was assessed in *M. auratus* hamsters infected with *Leishmania* (L.) *infantum chagasi*. The parasite burden in the liver and spleen was quantified by real-time PCR (Figure 4). Treatment with free or encapsulated LASSBio-1736 at doses up to 12.5 mg/kg/day reduced parasite load compared with untreated controls as well as the positive control (Glucantime, reference drug).

**Figure 4:**
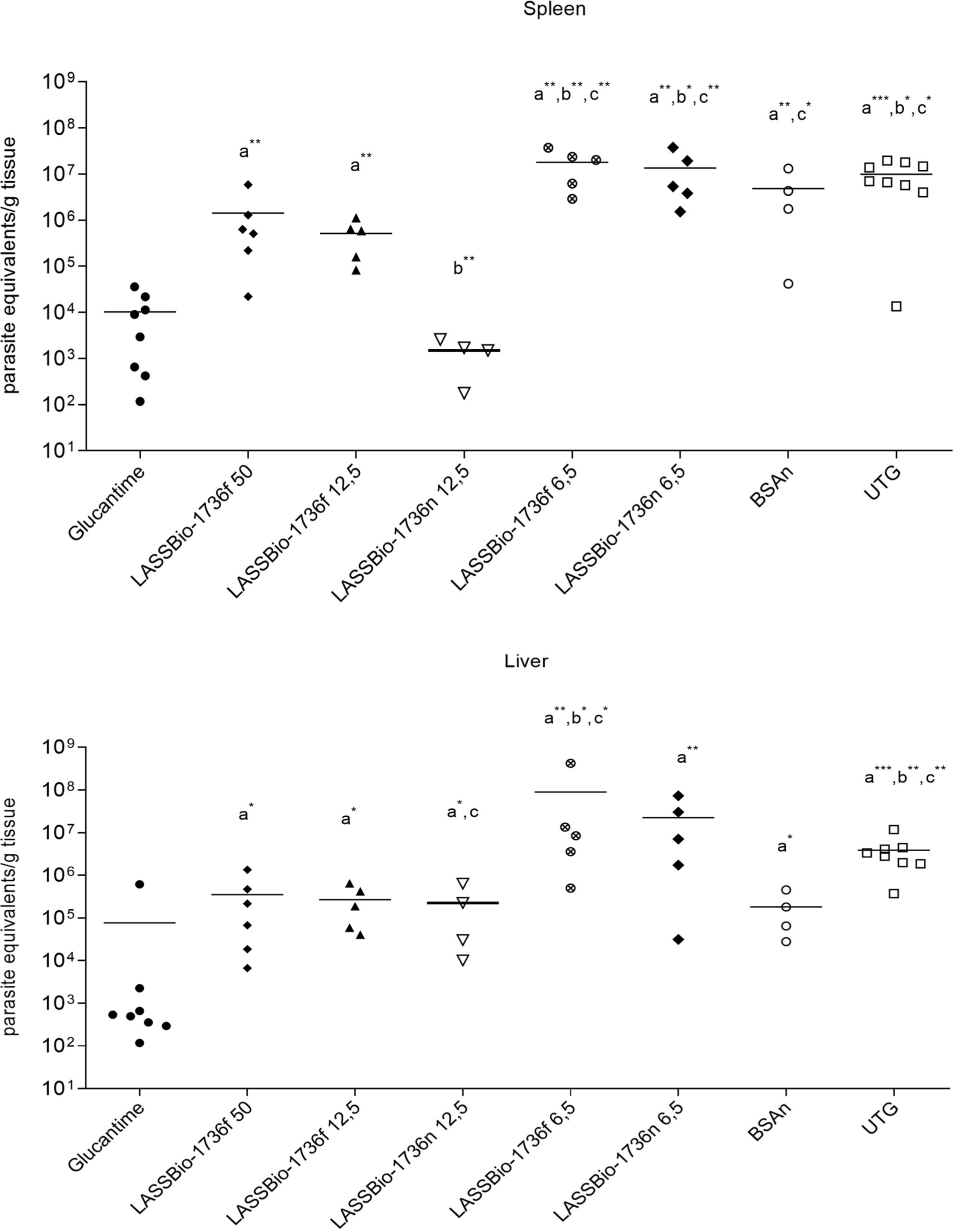
Parasite burden in the liver and spleen of hamsters infected with *Leishmania* (L.) *infantum chagasi* after treatment. Parasite load was quantified by quantitative PCR and expressed as parasite equivalents per gram of tissue. Each symbol represents an individual animal, and horizontal bars indicate median values for each group. Experimental groups included untreated infected animals (UTG), animals treated with LASSBio-1736f (free compound), animals treated with LASSBio-1736n (nanoparticle formulation), and animals treated with the reference drug meglumine antimoniate (Glucantime). Statistical comparisons between groups were performed using the Mann–Whitney test. Asterisks indicate statistically significant differences relative to the untreated group (*p < 0.05, **p < 0.01, and ***p < 0.001).

In addition, infected hamsters treated orally with LASSBio-1736n (12.5 mg/kg/day for 10 days) resulted in an 85.48% reduction in splenic parasite burden compared to Glucantime (*p* < 0.001). In contrast, LASSBio-1736n did not produce an additional reduction in hepatic parasite burden, whereas glucantime achieved a marked reduction in liver parasitism.

Gene expression levels of IFN-γ, IL-17, and IL-10 were evaluated in spleen and liver tissues (Figure 5). Glucantime induced the highest levels of both pro-inflammatory and regulatory cytokines, except for IL-17 in the liver. In contrast, treatment with encapsulated LASSBio-1736 and empty BSA nanoparticles resulted in lower IFN-γ and higher IL-10 expression in the liver, suggesting a more immunomodulatory rather than pro-inflammatory profile.

**Figure 5:**
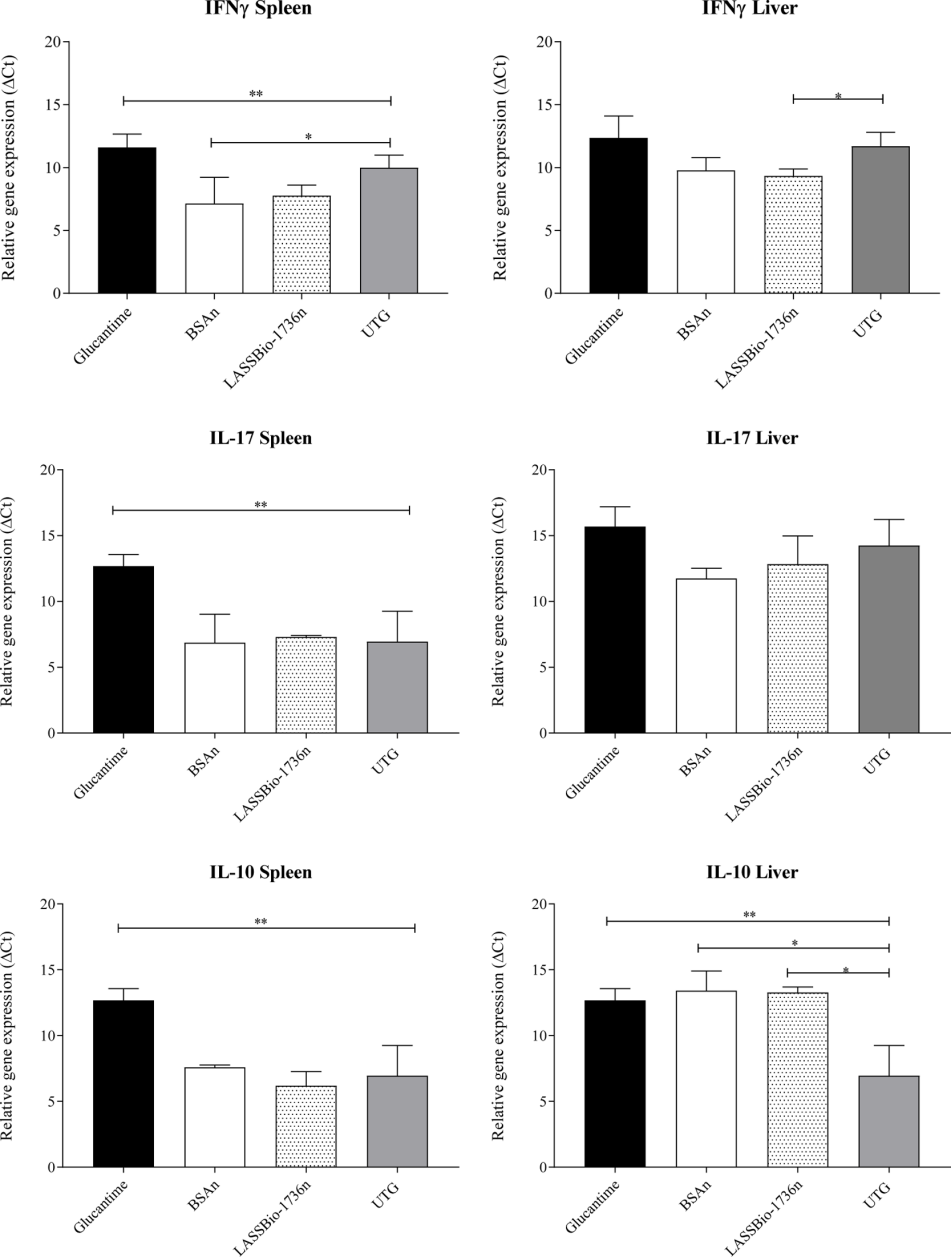
Relative mRNA expression of IFN-γ, IL-17, and IL-10 in the liver and spleen of hamsters after treatment. Gene expression levels were determined by quantitative PCR and expressed as relative mRNA expression (ΔCt). Animals were treated with LASSBio-1736n (12.5 mg/kg/day), empty BSA nanoparticles (BSAn), or remained untreated (UTG). Data are presented as mean ± SD (*n* = 3). Statistical comparisons between groups were performed using the Mann–Whitney test.

When cytokine expression (IFN-γ, IL-17, and IL-10) was analyzed in spleen and liver tissues of infected animals, the Glucantime group exhibited the highest overall levels, except for IL-17 in the liver. This treatment was associated with increased expression of both pro-inflammatory cytokines and IL-10, while maintaining better tissue integrity (data not shown). In contrast, nanoparticle-encapsulated LASSBio-1736 resulted in lower IFN-γ and higher IL-10 expression in the liver. Similarly, animals treated with empty BSA nanoparticles also exhibited increased IL-10 expression in the liver (Figure 5).

Glucantime (meglumine antimoniate) induced increased pro-inflammatory cytokines, particularly IFN-γ, consistent with parasite clearance.^23^ This response was accompanied by increased IL-10 levels, indicating a concurrent immunoregulatory response during *Leishmania* infection.^24^ Glucantime was administered parenterally at higher doses, whereas LASSBio-1736 formulations were given orally at lower doses, reflecting distinct pharmacological profiles.^3,20,21^ Thus, this comparison should be viewed as a translational benchmark rather than direct equivalence. Despite these differences, LASSBio-1736n markedly reduced splenic parasite burden, with a more balanced immunomodulatory profile.

In a vaccination study, reduced IL-10 expression was positively correlated with decreased parasite burden and lower liver damage in naturally infected dogs.^23^ Conversely, IL-10 plays an important role in modulating immune responses, maintaining homeostasis, and preventing tissue injury, particularly following the resolution of inflammatory stimuli.^24^

Histopathological analyses focused on collagen deposition, glycogen content, and the white-to-red pulp ratio in the spleen (Figure 6). Nanoparticulated LASSBio-treated animals exhibited increased hepatic glycogen, which is associated with improved tissue repair^25^ and better preservation of splenic white pulp compared with animals that received empty nanoparticles. Collagen distribution patterns supported the interpretation of enhanced tissue remodeling as previously described by Castro et al. (2018).^26^

**Figure 6:**
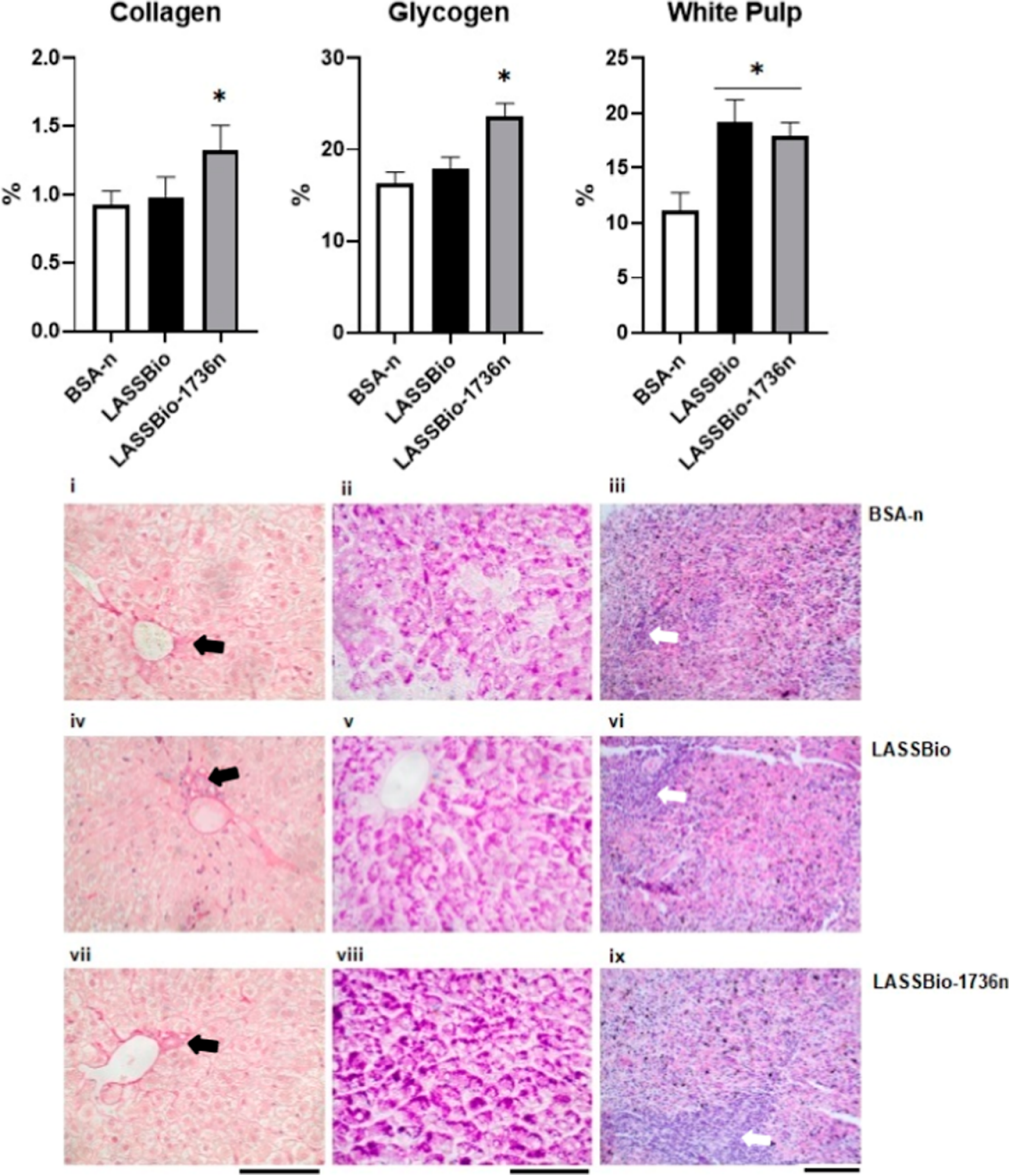
Histological analysis of liver and spleen after treatment. Collagen deposition (black arrows) in liver tissue was evaluated by Picro-Sirius Red (PSR) staining (graph 1 and images I, IV, and VII; bright field, ×400). Glycogen content in hepatic parenchyma (magenta-colored hepatocytes) was assessed by periodic acid–Schiff (PAS) staining (graph 2 and images II, V, and VIII; bright field, ×400). The white pulp ratio (white arrows) in spleen was evaluated by hematoxylin–eosin staining (graph 3 and images III, VI, and IX; bright field, ×200). Graphs show the quantitative analysis corresponding to the representative histological images. BSAn: empty BSA nanoparticles; LASSBio-1736n: BSA nanoparticles containing LASSBio-1736. The data were analyzed using one-way analysis of variance (ANOVA) followed by Tukey’s post hoc test.

Collagen deposition in the hepatic parenchyma is associated with two types of resident cells, particularly Kupffer cells and hepatic stellate cells (HSCs). In addition to promoting collagen production, these cells also contribute to the expression of acute-phase and antiapoptotic proteins that support tissue regeneration.^27^ HSCs also play a role in hepatocyte proliferation, which correlates with ploidy, as an increased percentage of binucleated cells is commonly observed in response to hepatic injury.^28,29^

Another process affected by liver injury is glycogen metabolism, which relies on the coordinated regulation of glycogen synthesis and degradation to maintain glucose availability and cellular energy homeostasis.^30^ Studies have shown suppression of glycolytic activity following hepatic damage.^31,32^ Glycogen metabolism plays a central role for energy supply during tissue regeneration, and increased hepatic glycogen content has been associated with improved outcomes.^33^ Moreover, some studies suggest that hepatic metabolism is closely linked to liver regeneration^34^ and that higher glycogen levels in the liver can reduce hepatic injury.^35^

In the splenic tissue, a significant difference in the white-to-red pulp ratio was observed between BSAn and LASSBio-1736n groups. The BSAn group showed hypoplasia and disorganization of the white pulp, a finding commonly reported in VL^36^ but attenuated in the LASSBio-1736n group.

Overall, these results demonstrate the ability of albumin-based nanocarriers to enhance the in vivo performance of LASSBio-1736n, supporting its development as an orally administered therapy for VL. The BSA nanoparticle platform provides multiple pharmacological advantages, including improved gastrointestinal stability, protection against enzymatic degradation, and enhanced intestinal permeability, ultimately increasing bioavailability. In addition, albumin-based nanocarriers exhibit intrinsic biological properties that facilitate drug transport and cellular targeting. Their natural affinity for albumin-binding proteins, such as gp60 and SPARC, enables receptor-mediated transcytosis and preferential accumulation in inflamed or macrophage-rich tissues, which are key sites of *Leishmania* infection. In this context, the improved in vivo performance of LASSBio-1736n may be attributed not only to enhanced bioavailability but also to these intrinsic targeting properties, with surface functionalization offering an additional potential for site-specific delivery optimization.^37^

In contrast to previous studies employing BSA-based nanocarriers, which commonly focus on repurposed or previously known compounds,^11^ the present work combines a rationally designed hydrazone derivative with a protein-based delivery system. This strategy enables simultaneous improvements in solubility, stability, and biological performance.^8–13^ Overall, this integrated approach highlights a more translationally relevant strategy for antileishmanial therapy.

Importantly, macrophage-targeted delivery systems have been shown to improve therapeutic outcomes in experimental leishmaniasis, as the *Leishmania* parasites reside within macrophages. Nanoparticles efficiently internalized by phagocytic cells could act as reservoirs that enable sustained intracellular drug release, increasing local concentrations at the site of infection while reducing off-target exposure.^38^ Such mechanisms likely underlie the favorable immunological and histopathological responses observed in infected tissues following treatment with LASSBio-1736-loaded BSA nanoparticles.

Beyond enhancing bioavailability and protecting against compound degradation, this nanocarrier system may also contribute to a reduced dosing frequency, an improved therapeutic index, and better patient compliance. Taken together, the combined pharmacokinetic and biological findings underscore LASSBio-1736n as a promising next-generation approach for safer and more effective oral therapy against VL. Further studies addressing long-term toxicity and in vivo pharmacokinetic behavior will be important to advancing the translational potential of this nanocarrier system.

## Materials and Methods

### Hydrazone Derivative

The hydrazone derivative (*E*)-1-(4-trifluoromethylbenzylidene)-5-(2,4-dichlorobenzoyl)­carbonohydrazide (LASSBio-1736) was kindly provided by Prof. Lídia Moreira Lima (INCT-INOFAR, LASSBio, Federal University of Rio de Janeiro) and synthesized as previously.^39^

### Preparation of BSA–LASSBio-1736 Nanoparticles

The empty nanoparticles were obtained by the addition of ethanol dropwise (ethanol/water ratio 1.5:1) in a solution of sterile 2% w/v BSA (Sigma-Aldrich catalog no. A2153, USA) in 10 mM NaCl (pH 8.5). The coacervates were hardened by adding 50 μL of 25% glutaraldehyde while stirring for 24 h at room temperature. The BSA nanoparticles were purified by three cycles of centrifugation at 13,000 g for 30 min to eliminate free BSA and the excess of the cross-linking agent. The supernatants were removed, and the pellets resuspended in sterile PBS (final concentration of 20 mg/mL). For the production of encapsulated LASSBio-1736 nanoparticles (LASSBio-1736n), 5 mg of LASSBio-1736 was dissolved in 500 μL of ethanol (10 mg/mL), and then the solution was mixed with 2 mL of 2% BSA solution (20 mg/mL). After gentle stirring, four drops of ethanol (∼5 μL each) and 17 μL of glutaraldehyde at 25% were added as a cross-linker, reaching a final volume of 2.23 mL and a theoretical drug concentration of 0.9 mg/mL. The suspension was centrifuged at 13,000*g* for 15 min as described above, the supernatants were discarded, and the nanoparticles were resuspended in PBS at 10 mg/mL.

### Nanoparticle Characterization

EE and drug LC were determined by ultrafiltration/centrifugation using Amicon Ultra 50 kDa filters. Free drug in filtrates and supernatants was quantified by HPLC-UV;^20^ and EE % and LC % were calculated from the difference between total and free drug. Particle size, PDI, and zeta potential were measured on a ZetaView PMX-430-Z (Particle Metrix, Germany) with 11 positions per sample (triplicates). Scanning electron microscopy was used to evaluate size and morphology. The produced BSA nanoparticles with or without LASSBio-1736 were characterized using SEM. The sample was drop-cast onto round glass coverslips (13 mm), dried in a desiccator at 50 °C for 1 h, and mounted on aluminum stubs. Then, samples were coated using a gold sputter coater (Denton Vacuum) for a coat of 20 nm of pure Au. Nanoparticles were observed and photographed using a scanning electron microscope (TESCAN-MIRA4 FEG) using an Everhart-Thornley Secondary Electrons Detector at 20 kV. Images were captured at the Centro de Microscopia da Universidade Federal de Alfenas, Minas Gerais, Brazil.

### In Vitro Gastrointestinal Stability (INFOGEST)

Gastrointestinal digestion was simulated according to the standardized INFOGEST protocol. LASSBio-1736n and Free-LASSBio-1736 (LASSBio-1736f) were sequentially exposed to simulated oral (pH 7, salivary fluid with amylase, 37 °C, 2 min), gastric (pH 3, gastric fluid with pepsin, 37 °C, 2 h), and intestinal (pH 7, intestinal fluid with bile salts and pancreatic enzymes, 37 °C, 2 h) phases, with and without enzymes. Samples collected at each phase were analyzed by ultra-high performance liquid chromatography tandem mass spectrometry (UHPLC-MS; model LCMS-2050, Shimadzu) using a methanol/formic acid (99:1, v/v) mobile phase at 0.2 mL/min and monitoring *m*/*z* 419. Relative stability was expressed as the percentage of compound remaining compared with enzyme-free controls.

### Intestinal Permeability (Caco-2 Assay)

Human Caco-2 cell monolayers (HTB-37, ATCC) were cultured on porous PTFE inserts until transepithelial electrical resistance exceeded 400 Ω·cm^2^. LASSBio-1736n and LASSBio-1736f (both 60 μM) were applied to the apical chamber for 30 min and 1 and 2 h. Samples from the basolateral chamber were analyzed by HPLC-UV, and apparent permeability (P_app) was calculated as previously described.^20^

### In Vivo Leishmanicidal Evaluation

Female golden hamsters (*M. auratus*, 80–100 g) were housed under controlled conditions with free access to food and water. After 8 weeks of age, animals were infected intraperitoneally with 2 × 10^7^ amastigotes of *Leishmania* (L.) *infantum chagasi* (MHOM/BR/1972/BH46), prepared from spleens of previously infected donors. Sixty days after infection, animals were randomized into treatment groups and received oral therapy for 10 days as follows: untreated controls; Glucantime and LASSBio-1736f at 50 mg/kg/day; and LASSBio-1736f or LASSBio-1736n at 6.25 or 12.5 mg/kg/day. All animal procedures were approved by the Institutional Animal Care and Use Committee (n°. 0031–2021).

Parasite burden in spleen and liver was determined by quantitative PCR,^40^ as well as cytokine expression (IL-10 and IFN-γ).^41^ Histopathological analysis of liver and spleen was performed in paraformaldehyde-fixed and paraffin-embedded tissues.^42,43^ For analysis of collagen and glycogen content, liver tissues were stained with PSR and PAS, according to Dos Reis et al. (2024),^25^ reported as described by Mendonça et al., 2020.^44^ Hematoxylin–eosin staining was used on spleen slides to analyze white pulp/red pulp ratio.^45^

### Statistical Analysis

All experiments were performed at least in triplicate. Data are expressed as mean ± SD. Data normality was assessed prior to statistical testing. Parametric data were analyzed using one-way ANOVA followed by Tukey’s post hoc test. Nonparametric data were analyzed using the Mann–Whitney test. The specific statistical test applied to each data set is indicated in the corresponding figure legends. A *p*-value <0.05 was considered statistically significant.

## Supplementary Material



## Notes

HPLC calibration curves for EE, drug LC, UHPLC–MS quantification, and Caco-2 permeability analysis; residual compound quantification after simulated gastrointestinal digestion (PDF)
