# Supplementary material for: Leishmanicidal Activity of a Hydrazone Derivative Loaded into Nanocarrier Systems
Source: ACS Omega. 2026 May 27;11(22):32242–51. doi: 10.1021/acsomega.5c13239 (PMC13261406; doi:10.1021/acsomega.5c13239)
Supplement: Supplementary file 1 [file ao5c13239_si_001.pdf]

# LEISHMANICIDAL ACTIVITY OF A HYDRAZONE DERIVATIVE LOADED INTO NANOCARRIER SYSTEMS

Juliana B. Nunes[a], Thalles H. F. de Souza[a], Amanda S. Lima[b], Clara O. C. Lopes[c], Isabelly F. Ferraz de Souza[c], Raíne P. Amaral[d], Gislaine R. Pereira[c], Fábio A. Colombo[c], Eduardo C. Figueiredo[c], Luciana Azevedo[b], Luiz F. Leomil Coelho[d], Luis F. Cunha dos Reis[e*], Lídia M. Lima[f], Marcos J. Marques[a*]

Corresponding authors

*Biomedical Sciences Institute, Structural Biology Department, Alfenas Federal University (UNIFAL-MG), 37.130-001 Alfenas, Brazil. luis.cunha@unifal-mg.edu.br

*Biomedical Sciences Institute, Pathology and Parasitology Department, Alfenas Federal University (UNIFAL-MG), 37.130-001 Alfenas, Brazil. marcos.marques@unifal-mg.edu.br

[a]Biomedical Sciences Institute, Pathology and Parasitology Department, Alfenas Federal University (UNIFAL-MG), 37.130-001 Alfenas, Brazil.

[b]Nutrition School, Alfenas Federal University (UNIFAL-MG), 37.130-001 Alfenas, Brazil.

[c]School of Pharmaceutical Sciences Alfenas Federal University (UNIFAL-MG), 37.130-001 Alfenas, Brazil.

Brazil.

[d]Biomedical Sciences Institute, Microbiology and Immunology Department, Alfenas Federal University (UNIFAL-MG), 37.130-001 Alfenas, Brazil. Alfenas Federal University (UNIFAL-MG), 37.130-001 Alfenas, Brazil.

[e]Biomedical Sciences Institute, Structural Biology Department, Alfenas Federal University (UNIFAL-MG), 37.130-001 Alfenas, Brazil. Alfenas Federal University (UNIFAL-MG), 37.130-001 Alfenas, Brazil.

[f]Faculty of Pharmacy, Federal University of Rio de Janeiro (UFRJ), Av. Carlos Chagas Filho, 373, Cidade Universitária, Rio de Janeiro, RJ 21941-902, Brazil.

## Supporting Information

Optimization curve of LASSBio-1736 by HPLC (Figure S1); calibration curve after *in vitro* digestion (Figure S2); calibration curve for permeation experiments (Figure S3); residual concentrations after INFOGEST digestion (Table S1).

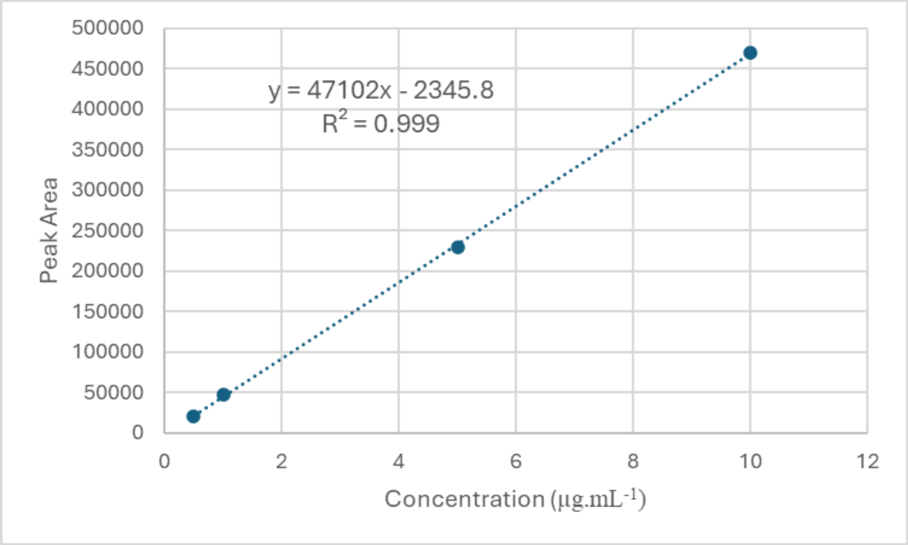


Figure S1 – Optimization curve of the compound LASSBio-1736 by HPLC.

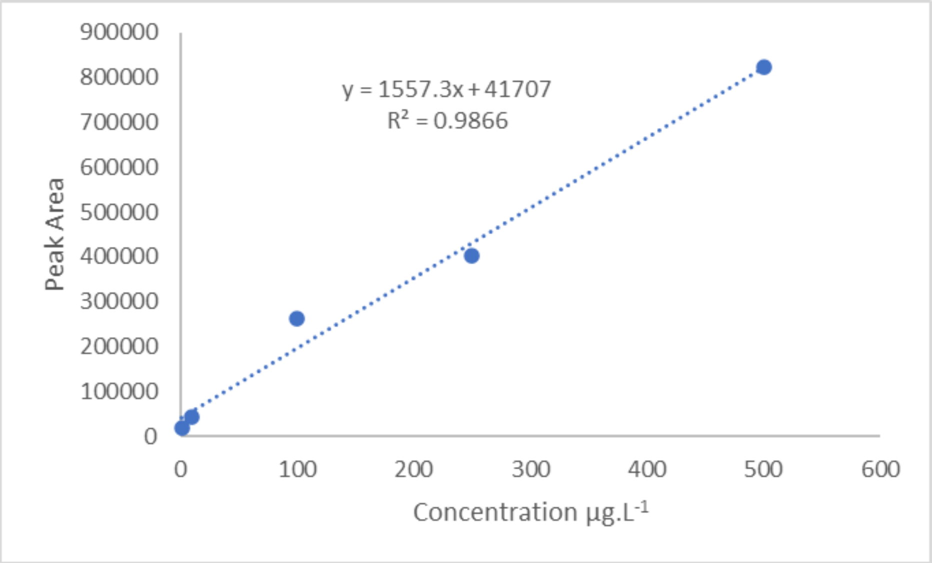


Figure S2 – Calibration curve used to quantify LASSBio-1736 after *in vitro* digestion.

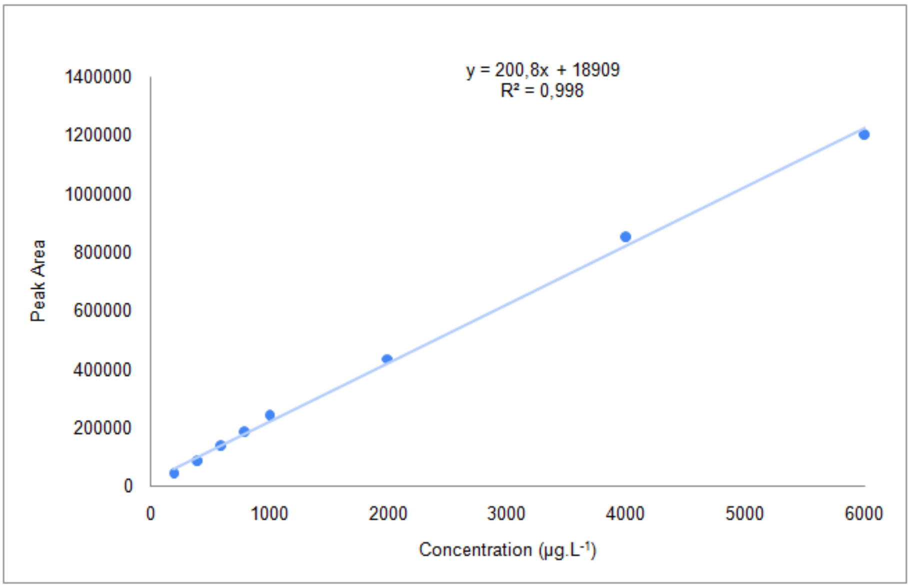


Figure S3 – Calibration curve used to quantify LASSBio-1736 in the permeation experiment.

Table S1 – Residual concentration of LASSBio-1736 after INFOGEST digestion, calculated by UHPLC–MS.

| Phase/Compound | Concentration (µg/mL) | Ratio C/S | % Weighted* |
|---|---|---|---|
| FOPS | 6.0 | 110.6798093 | 35.4 |
| FOPC | 6.6 | | |
| FONS | 10.3 | 43.76759244 | 14.0 |
| FONC | 4.5 | | |
| FGPS | 22.4 | 43.12 | 13.8 |
| FGPC | 9.7 | | |
| FGNS | 14.7 | 91.99 | 29.44 |
| FGNC | 13.5 | | |
| FIPS | 101.2 | 85.7 | 27.41 |
| FIPC | 86.7 | | |
| FINS | 21.8 | 312.45 | 100.0 |
| FINC | 68.1 | | |

FO – oral phase; FG – gastric phase; FI – intestinal phase; P – LASSBio-1736;

N – nanoparticulated LASSBio-1736; S – without enzyme; C – with enzyme

*Statistical difference $p < 0.00001$
